# T Cells Contribute to Stroke-Induced Lymphopenia in Rats

**DOI:** 10.1371/journal.pone.0059602

**Published:** 2013-03-15

**Authors:** Lijuan Gu, Xiaoxing Xiong, Dingtai Wei, Xuwen Gao, Sheri Krams, Heng Zhao

**Affiliations:** 1 Institute of Immunology, Zhejiang University School of Medicine, Hangzhou, China; 2 Department of Neurosurgery, Stanford University School of Medicine, Stanford, California, United States of America; 3 Department of Surgery, Stanford University School of Medicine, Stanford, California, United States of America; University of Amsterdam Academic Medical Center, The Netherlands

## Abstract

Stroke-induced immunodepression (SIID) results when T cell and non-T immune cells, such as B cells, NK cells and monocytes, are reduced in the peripheral blood and spleen after stroke. We investigated the hypothesis that T cells are required for the reductions in non-T cell subsets observed in SIID, and further examined a potential correlation between lymphopenia and High-mobility group protein B1 (HMGB1) release, a protein that regulates inflammation and immunodepression. Our results showed that focal ischemia resulted in similar cortical infarct sizes in both wild type (WT) Sprague Dawley (SD) rats and nude rats with a SD genetic background, which excludes the possibility of different infarct sizes affecting SIID. In addition, the numbers of CD68-positive macrophages in the ischemic brain did not differ between WT and nude rats. Numbers of total peripheral blood mononuclear cells (PBMCs) or splenocytes and lymphocyte subsets, including T cells, CD4^+^ or CD8^+^ T cells, B cells and monocytes in the blood and spleen, were decreased after stroke in WT rats. In nude rats, however, the total number of PBMCs and absolute numbers of NK cells, B cells and monocytes were increased in the peripheral blood after stroke; nude rats are athymic therefore they have few T cells present. Adoptive transfer of WT splenocytes into nude rats before stroke resulted in lymphopenia after stroke similar to WT rats. Moreover, *in vitro* T cell proliferation stimulated by Concanavalin A was significantly inhibited in WT rats as well as in nude rats receiving WT splenocyte adoptive transfer, suggesting that T cell function is indeed inhibited after stroke. Lastly, we demonstrated that stroke-induced lymphopenia is associated with a reduction in HMGB1 release in the peripheral blood. In conclusion, T cells are required for stroke-induced reductions in non-T immune cells and they are the most crucial lymphocytes for SIID.

## Introduction

Stroke-induced immunodepression (SIID) results in infection, which is considered to be the major complication leading to delayed mortality in stroke patients [1], [2], [3], [4]. A SIID hallmark is lymphopenia, which is characterized by decreased immune cells in the peripheral blood and spleen [4], [5]. Research into SIID dates back to more than 40 years [6], when it was found that death after stroke was more frequently associated with non-neurologic diseases, such as pneumonia, pulmonary embolism and urinary tract infections. Thereafter, strong evidence from clinical studies has shown that stroke causes a reduction of T cells in the peripheral blood and inhibition of T cell proliferation in response to antigen stimulation, as well as inhibition of the delayed type hypersensitivity (DTH) skin test [7]. Most studies agree that T-cell-mediated immunity is significantly inhibited by brain injury from stroke [7], [8], [9], [10], [11]. However, there are contradictory reports about the effects of stroke on humoral immunity in human patients. For example, Urra et al. reported that B cells in stroke patients were reduced [12], whereas Vogelgesang et al. reported they were unchanged [10]. In addition, an early study of stroke patients suggests that IgM and IgG immunoglobulins were unchanged but IgA was increased [7].

In recent years several groups have used animal models to confirm in principle the phenomenon of SIID and to understand the underlying mechanisms involved [5], [13], [14], [15], [16]. In a mouse stroke model, Prass et al. found that B cells, T cells and NK cells were reduced in the spleen and peripheral blood, which may have resulted from the elevated lymphocyte apoptosis observed in the spleen and thymus [4]. In addition, cytokine expression shifted from a pro-inflammatory Th1 profile to an anti-inflammatory Th2 profile in the peripheral lymphoid organs [4]. They further demonstrated that sympathetic nervous system (SNS) activation plays a critical role in SIID [4]. Most recently, Wong and colleagues reported that stroke led to the activation of the SNS, which innervates iNKT cells in the liver, causing iNKT cells to secrete immunosuppressive cytokine IL-10, rather than pro-inflammatory IFNγ, thus resulting in SIID [17]. Alternatively, Offner and colleagues suggest that SIID might be induced by increases in regulatory T cells (Tregs) after stroke while other T cell subsets, such as CD4^+^ and CD8^+^ T cells, as well as B cells were reduced [5].

Despite these pioneer studies, issues still remain. First, although nearly every study reported that T cells were reduced after stroke, whether or not B cells [5], [18], NK cells [4], [10], [19] and monocytes [5], [11], [20], [21] in the peripheral organs are also reduced remains controversial among clinical studies and mouse stroke studies. More studies from other animal models, such as rat stroke models, may help to clarify these issues. Second, it is well known that cell-mediated and humoral immunity cross-react as does adaptive and innate immunity. We hypothesized that T cells play a pivotal role in determining the fates of other non-T immune cells and investigated SIID in a rat stroke model. We used T-cell-deficient nude rats to further address how T cell deficiency affects lymphopenia in other cell types after stroke. Third, as the cytokine-like protein, High-mobility group protein B1 (HMGB1), is released into the blood after stroke [22], [23], and HMGB1 is known to be involved in both inflammation [23], [24], [25] and immunodepression [26], we examined if HMGB1 release in the plasma correlates with T cells and lymphopenia, and studied the effects of a HMGB1 inhibitor, glycyrrhizin [27], on lymphopenia after stroke.

## Materials and Methods

All studies were conducted in accordance with protocols approved by the Institutional Animal Care and Use Committees at Stanford University and according to the NIH Guide for Care and Use of Laboratory Animals.

### Generation and genotyping of nude rats

Male nude rats (NCI, Frederick, MD, USA) and female Sprague Dawley (SD) rats (Charles River, Wilmington, MA, USA) were group-housed under standard laboratory conditions and kept on a normal light/dark cycle. A male nude rat was backcrossed to female SD rats to generate heterozygous (rnu/+) rats. The heterozygous rats were then backcrossed to female SD rats for at least eight generations. The siblings of heterozygous were mated to generate homozygous (rnu/rnu) nude rats with a SD genetic background. Genotyping was determined by PCR and subsequent restriction fragment length polymorphism (RFLP) as described previously [28]. In brief, genomic DNA was extracted from the tail of rats using a DNeasy Blood and Tissue Kit (Qiagen, Germantown, MD, USA), and genomic PCR was performed with Taq DNA Polymerase High Fidelity (Invitrogen, Carlsbad, CA, USA) under the following conditions: 95°C for 30 s; 30 cycles at 94°C for 15 s, 55°C for 15 s, and 72°C for 35 s; and 72°C for 5 min. Forward PCR primers were 5′-CACCAGCAGCCATTGTTGTCA-3′ and reverse primers were 5′-CATGGTCCTGGCTGAGGAAG-3′. PCR products were digested by restriction endonuclease Tsp45i (New England Biolabs, Ipswich, MA, USA) at 65°C for 1 h. Digested products were then analyzed by electrophoresis on 4% agarose gels with ethidium bromide.

### Focal cerebral ischemia and infarct size measurement

WT and nude rat littermates were used at 10–12 weeks of age (280–320 g). Focal ischemia in male SD rats and T-cell-deficient nude rats was performed as described previously [29], [30]. In brief, anesthesia was induced by 5% isoflurane and maintained with 2–3% isoflurane during surgery. Core body temperatures were monitored with a rectal probe and maintained at 37°C throughout the experiment. The distal middle cerebral artery (dMCA) was exposed and permanently cauterized above the rhinal fissure. The bilateral common carotid arteries (CCAs) were occluded for 1 h. The CCAs were then released and the wound was sutured. Rats were euthanized with an overdose of isoflurane 3 days post-stroke. Brains were removed and sectioned coronally at 2 mm intervals and stained in a 2% solution of 2,3,7-triphenyltetrazolium chloride (TTC). Using a computerized image analysis system (NIH image, version 1.61), the infarct area of 5 sections was measured. The infarct size of the ischemic cortex was normalized to the non-ischemic cortex and expressed as a percentage, as described previously [29], [30].

### Immunofluorescent staining

Sham-operated WT rats and ischemic WT or nude rats were euthanized with an overdose of isoflurane and perfused with icy PBS, followed by 4% paraformaldehyde in phosphate-buffered saline (PBS) (pH 7.4) 72 h after stroke onset as described previously [31]. The brains were dissected out and fixed in 4% paraformaldehyde in PBS (pH 7.4) for 48 h and sliced into 50 μm sections. Free-floating sections were used for immunofluorescent staining under moderate shaking. All buffers for washes and incubations were 0.1 M PBS (pH 7.4) containing 0.3% triton X-100. Sections were incubated overnight at 4°C with a mouse anti-rat CD68 primary antibody (diluted 1∶200; MCA341GA, AbD Serotec, Oxford, UK) after being blocked for 1 h with blocking solution (0.1 M PBS, 0.3% Triton X-100 and 5% equine serum). The sections were then rinsed and incubated for 2 h at room temperature with Alexa 488-conjugated goat anti-mouse IgG (diluted 1∶200, Invitrogen, Carlsbad, CA, U.S.A). Then, the sections were washed and mounted on glass slides using a Vectashield mounting medium with 4′, 6-diamidino-2-phenylindole (DAPI; Vector Laboratories, Burlingame, CA, U.S.A). Negative controls without primary antibodies were performed in parallel. The expression of CD68 was evaluated by using the optical fractionator method on epifluorescent photomicrographs (Zeiss axiovert inverted scanning microscope; Zeiss, Oberkochen, Germany) covered a total of 0.14 mm^2^. Three sections were randomly chosen for an average value in each rat, and 4–5 animals were studied in each group. The number of CD68 immunoreactive cells in the predesigned infarct area was counted using Image J software (Image J 1.37v; Wayne Rasband, available through National Institutes of Health). All counts were performed blindly to the investigator on coded sections [31].

### Isolation of mononuclear cells from the brain, blood and spleen

Rats were deeply anesthetized and 1–1.5 ml peripheral blood from the tail was collected in a heparinized tube. Rats were then perfused transcardially with 200 ml cold PBS, and spleens were harvested in tissue culture dishes containing 20 ml cold RPMI 1640 (Invitrogen, Carlsbad, CA, USA). Brains were collected and mononuclear cells were isolated as described by Lim et al. [32]. Both ipsilateral and contralateral cortices were dissected and homogenized with the FACS buffer (F-PBS, PBS with 1% FBS and 0.1% sodium azide). After centrifugation, the cells were resuspended in 7 ml FACS buffer, completely mixed with 3 ml 90% Percoll (GE Healthcare, Pittsburgh, PA, USA), and 1 ml of 70% Percoll (in PBS) was loaded under the cell suspension. The cell suspension was then centrifuged at 2470 rpm for 30 min at 4°C and mononuclear cells at the interphase were collected. After washed in FACS buffer, cells were filtered through a 70 μm strainer and re-suspended in 1 ml FACS buffer, then counted.

The blood collected 48 or 72 h post-stroke was centrifuged at 1400 rpm. The resulting cell pellet containing the PBMCs was resuspended in 15 ml RPMI 1640 (Invitrogen, Carlsbad, CA, USA) and slowly underlaid with 13 ml Ficoll-Paque PLUS (GE Healthcare, Pittsburgh, PA, USA). This solution was centrifuged at 2200 rpm without break for 30 min at room temperature. The intermediate cloudy cell layer was transferred to a new 50 ml Falcon tube, resuspended in RPMI 1640 medium and centrifuged at 1400 rpm for 10 min at room temperature. The PBMCs were resuspended in 2 ml RPMI 1640 and counted.

To isolate the splenocytes, spleens harvested 72 h post-stroke were chopped into small pieces, homogenized by the plunger tip of a 3 ml syringe, filtered through a 70 μm strainer and centrifuged at 1200 rpm for 5 min. After erythrocytes were lysed using ACK lysing buffer (GIBCO, Invitrogen, Carlsbad, CA, USA), the cells were washed twice and resuspended in 20 ml RPMI1640 medium and counted.

### FACS analysis for immune cell populations

Mononuclears were labeled with mAbs for membrane expression of TCR, NKRP, SIRP, CD4, CD8, CD25, CD45RA and intracellular Foxp3. All primary antibodies were purchased from AbD Serotec (AbD Serotec, Oxford, UK), except CD8a-PE (BD Pharmingen, San Diego, CA, USA) and Foxp3-FITC (eBioscience, San Diego, CA, USA). The mouse/rat Foxp3 staining kit (eBioscience) was used to detect Treg levels. RPE-Cy5-, PE-, and FITC-conjugated mouse IgG1, rIgG2a and IgG2b were used as isotype matched controls. Briefly, Cells were washed once with 1 ml cold FACS buffer (PBS with 1% FBS and 0.1% sodium azide), and incubated for 20 min on ice with primary antibodies. Cells were then washed for a second time in 1 ml FACS buffer and incubated with secondary antibodies for 20 min on ice, as needed. The cells received a final wash with 2 ml FACS buffer.

For intracellular staining of Foxp3, 1 ml of freshly prepared Fix/Perm buffer was added to each sample tube. Cells were incubated in darkness at room temperature for 30 min, washed twice with 2 ml freshly prepared permeabilization buffer, and centrifuged. Next 1 μl of anti-mouse/rat Foxp3-FITC mAbs was added to each tube and incubated in darkness at room temperature for 30 min, washed twice with 2 ml permeabilization buffer.

Stained Cells were resuspended in 400 μl of FACS buffer for analysis by flow cytometry using BD FACscan and CELLQuest software (Becton Dickinson, San Jose, CA, USA). FlowJo version 7.6.2 was used to analyze the cell populations.

### Glycyrrhizin administration

Glycyrrhizin (0.30 g, Calbiochem, Millipore, MA, USA), an HMGB1 inhibitor, was dissolved in 1.5 ml 50 mM NaOH. PH value was titrated to 7.0 with 0.5 M HCl. Then 1 ml 10× Tris buffer (pH  = 7.5) was added and distilled water was supplied to reach a total 10 ml (30 mg/ml). Glycyrrhizin was injected intraperitoneally immediately before MCAO onset and 2 h after reperfusion at a dose of 200 mg/kg. Same volume of vehicle was injected as control. Animals were euthanized 3 d post stroke. PBMCs were isolated and counted as described above.

### Splenectomy

Rats were deeply anesthetized with isoflurane. Splenectomy was conducted as described previously with revision [33]. In brief, a 2-cm dorsal midline skin incision was cut at the caudal terminus along the level of the 13th rib. The spleen containing blood vessels was removed through the incision by using blunt forceps. The blood vessels were cauterized. Then, focal ischemic stroke was performed immediately after the abdominal wall and incision were sutured. Rats were allowed for 48 h survival.

### Western Blotting for HMGB1 release in the peripheral blood

Rats were deeply anesthetized 2 d after stroke and 1.5 ml peripheral blood from the heart was collected in heparinized tube and mixed well. Then, the blood was centrifuged at 1500× g for 10min at room temperature. Plasma, the top yellow supernatant fluid, was collected in a new Eppendorf tube. 50 ul of 5X SDS was added into 200 μl of sample. The samples were then heated for 5 min at 90°C and then stored at −20°C. Western blot was performed as described with modification [30], [34], [35]. In each lane, 15 μl of sample was loaded to sodium dodecyl sulfate – polyacrylamide gel electrophoresis by using 4–15% Ready Gel (Bio-Rad Laboratories, Hercules, CA, USA) for 1 h. Proteins were transferred to polyvinylidene fluoride (Millipore, Bedford, MA, USA) membranes for 1 h. The membrane was blocked with 5% nonfat dry milk (Bio-Rad Laboratories) in PBS containing 0.05% Tween-20 (Sigma, Santa Clara, CA, USA) (PBST) for 1 h and incubated with primary rabbit anti-HMGB1 antibody (diluted 1∶500, ab18256, Abcam Inc, MA, USA) overnight at 4°C, and followed by 1 h of incubation with a goat anti-rabbit IgG conjugated by horseradish peroxidase (HRP) (1∶3,000, Cell Signaling Technology, Danvers, MA, U.S.A). Then, membranes were scanned using Typhoon trio (GE Healthcare).

### Adoptive splenocyte transfer for reconstitution of T cells in nude rats

Naïve Sprague Dawley rats were deeply anesthetized and euthanized according to a protocol approved by the Stanford University Animal Care and Use Committee. Splenocytes were isolated as described above. Cell concentration was then adjusted to 10^9^ splenocytes/ml in RPMI 1640 and 100 μl was immediately administered intravenously via the external jugular vein to nude rats. Stroke was performed 24 h after reconstitution.

### T cell proliferative assay

Splenocytes were harvested 72 h after stroke onset. Cells were adjusted to 2×10^6^/ml, and 100 μl/well was loaded into 96 well tissue culture plates and incubated at 37°C, 5% CO2 for 66 h in the presence of 1 μg/ml T cell mitogen Concanavalin A (Con A, Sigma-Aldrich, Santa Clara, CA, USA). [3H]-thymidine (PerkinElmer, Waltham, MA, USA) was then added to the cells and incubation was continued for another 18 h. Cells were collected onto a glass fiber filter (PerkinElmer, Waltham, MA, USA). Thymidine uptake was determined by liquid scintillation in a 1250 β-plate scintillation reader (PerkinElmer, Waltham, MA, USA). Results are expressed as counts per minute (cpm). Each sample was in triplicate in each plate, and repeated at least 3 times on different days.

### Statistical Analysis

All data were normally distributed, which was tested with One-Sample Kolmogorov-Smirnov Test by using SPSS 16.0. Asymptotic significance level >0.05 was considered to meet Normal distribution. One-way analysis of variance (ANOVA) was used to compare each group of cell populations followed by the Fisher *post hoc* test when there were more than 3 groups; the t-test was used on only 2 groups to compare infarct sizes. Tests were considered significant at p-values <0.05. Data are presented as mean ± SEM.

## Results

### Successful generation of nude rats with a Sprague Dawley genetic background

**Figure 1:**
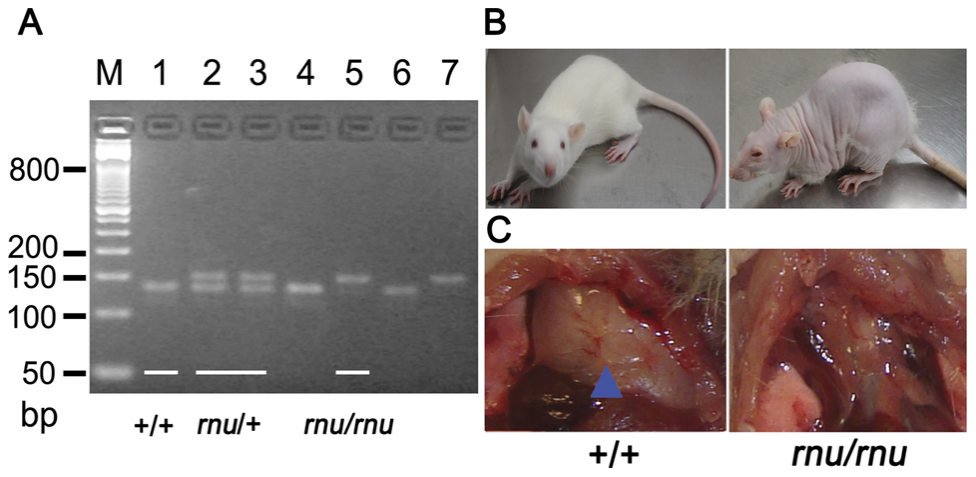
PCR-RFLP assay for genotyping SD-rnu rats. A. The PCR products were analyzed on 4% agarose gel. Lane M: DNA size marker; Lane 1, 4, 6: SD homozygote (+/+); Lane 2, 3: heterozygote (rnu/+); Lane 5, 7: nude homozygote (rnu/rnu). B. Representative appearance of WT SD rat and nude rat without hair. C. The WT SD rat has a thymus (arrow) while the nude rat is athymic.

In order to study that the effects of stroke on peripheral immune cells in nude and WT rats are due to T cell deficiency rather than to different genetic backgrounds, nude rats carrying the SD genetic background were bred as described in the Materials and Methods section. Three genotypes were clearly distinguished from each other after Tsp45i digestion of the PCR products: normal homozygote (+/+), heterozygote (+/rnu) and nude homozygote (rnu/rnu). Rats with (+/+) and (+/rnu) genotypes have normal thymuses, while (rnu/rnu) genotype rats are athymic (Fig. 1). T cells differentiate, develop and mature in the thymus; the generated athymic nude rats are therefore T-cell-deficient with a SD genetic background.

### Infarct sizes and activated macrophages in the ischemic brain did not differ between wild type and nude rats

**Figure 2:**
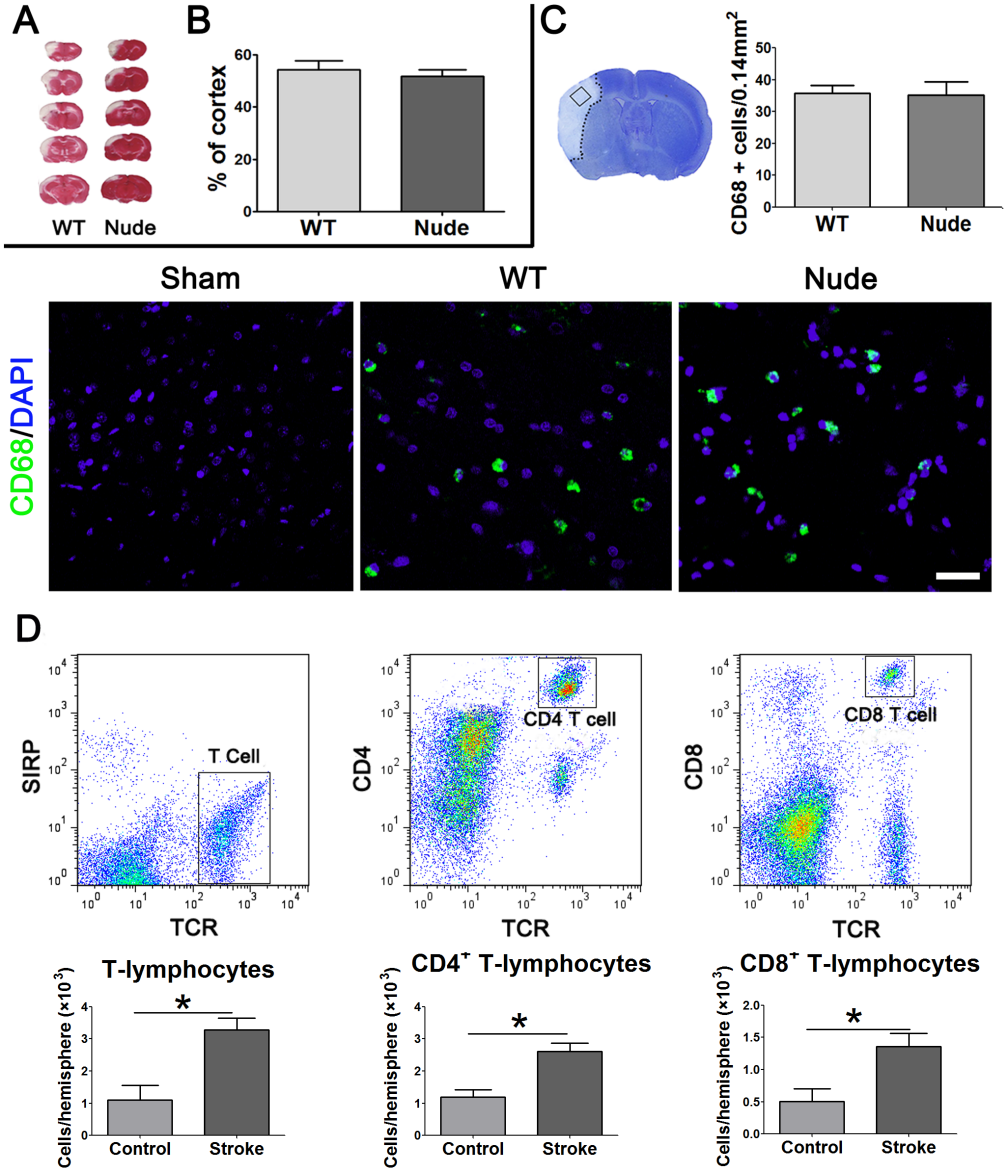
Cortical infarction and inflammation in WT and nude rats after stroke. A. Representative infarctions in the cortex are shown. Animals were euthanized 3 d after stroke. Brains were collected, coronally sectioned into 5 slices and stained with a 2% TTC solution. B. Infarct regions were measured and normalized to the contralateral, non-ischemic cortex, and expressed as percentage. The bar graphs represent average infarct sizes (n = 7–9/group). C. CD 68-positive macrophage staining in the ischemic brain. Ischemic brains were collected 72 h post-stroke and immunostained with CD 68 antibodies (green), and counterstained with DAPI (blue). CD 68 positive cells were counted from 3 sections of each animal. Representative images of CD68 positive macrophages are shown. The statistical results suggest no difference in the CD 68 positive macrophage numbers between WT and nude rats. N = 4–5/group. Scale Bar: 50 μm. D. Ischemic brain cortices were collected 3 d after stroke and total mononuclear cells were extracted for FACS analyses. Data from the non-ischemic hemispheres were used as controls. Gating strategy for T cells and subpopulations are shown on the upper panel. In the ischemic brain, numbers of T cells and CD4^+^ T cells, as well as CD8^+^ T cells were significantly increased compared with those in the control hemisphere. Note that nude rats, in principle, have no T cells, including CD4^+^ T cells, CD8^+^ T cells. * vs. corresponding controls (the contralateral, non-ischemic hemisphere), *P*<0.05, n = 4.

Stroke generated by the distal MCA occlusion (dMCAO) model results in robust cortical infarction in both WT and T-cell-deficient nude rats (Fig. 2A–B). Statistical analyses suggest there were no differences between infarct sizes of WT and nude rats. As infarct size and location may affect the degree of SIID in WT rats alone, this result indicates that the potential difference in SIID between WT and nude rats revealed in this study is due to T cells rather than infarct size or location.

To compare inflammatory conditions in the ischemic brains between WT and nude rats, we performed immunostaining of CD68, an activated macrophage marker. The results showed that no positive staining was detected in non-ischemic brain, but CD68-positive cells were increased in both ischemic brains of WT and nude rats 72 h post-stroke (Fig. 2C). Between WT and nude rats, there was no significant difference in the number of CD68-positive cells in the ischemic area (Fig. 2C). We then investigated the lymphocytes infiltration in the brain 3 d after stroke by FACS analysis. The gating strategies used to analyze lymphocyte subsets are shown (Fig. 2D). The results showed that T cells, CD4^+^ and CD8^+^ T cells were significantly increased in the ischemic hemisphere compared with the non-ischemic brain (Fig. 2D).

### Stroke produced lymphopenia in WT rats but not in T-cell-deficient nude rats

**Figure 3:**
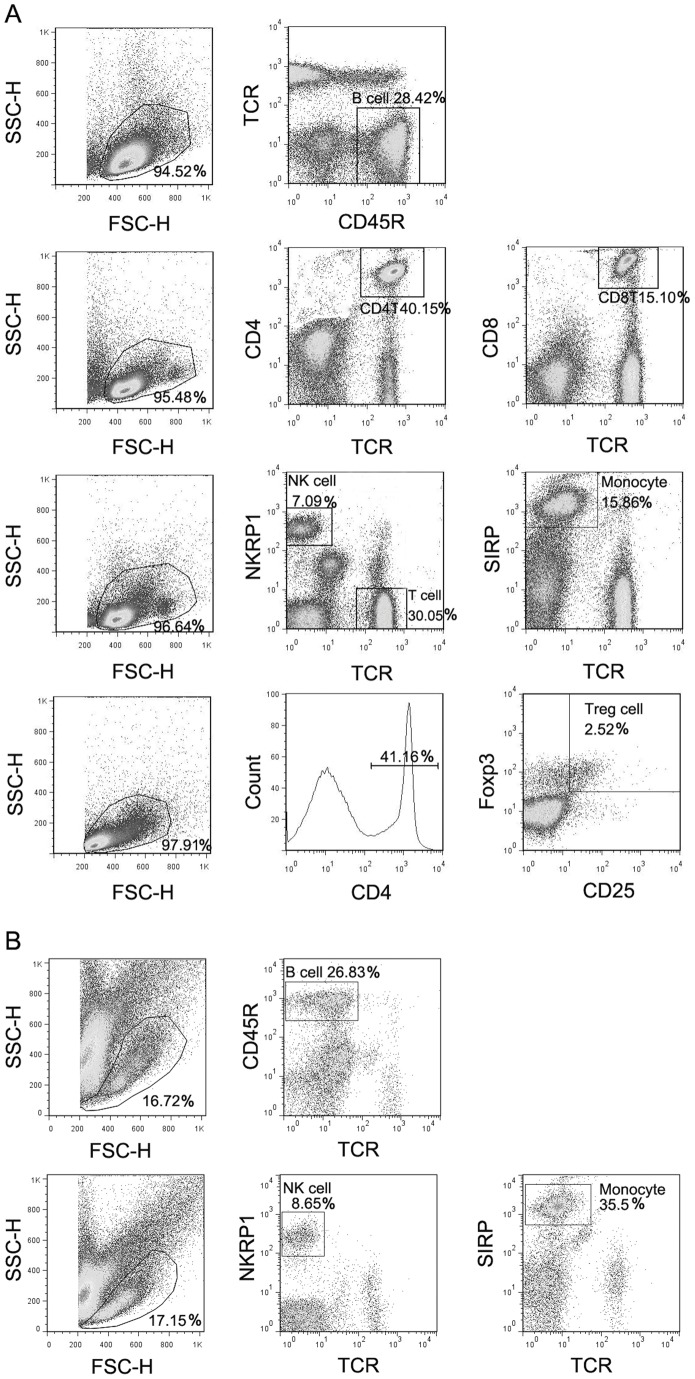
Gating strategy for immune cell subpopulations in the blood and spleen. Blood was harvested from the tail vein 3 d after stroke and total PBMCs were isolated for immunostaining. The first column in each row shows the gated total PBMCs based on Forward Scatter (FSC) and Side Scatter (SSC) parameters. Further analyses after immunofluorescent staining for specific cell populations are shown in columns 2 and 3. The relative percentage of the gated population is shown at the corner. Cell populations are defined as: B cells, CD45R^+^TCR^−^; CD4^+^ T cells, CD4^+^TCR^+^; CD8^+^ T cells, CD8^+^TCR^+^; macrophages, SIRP^+^TCR^−^; NK cells, NKR^+^TCR; Tregs, CD4^+^CD25^+^FoxP3^+^. A. Representative FACS profiles after gating to detect cell types in WT rats. B. Representative FACS profiles after gating to detect cell types in nude rats.

**Figure 4:**
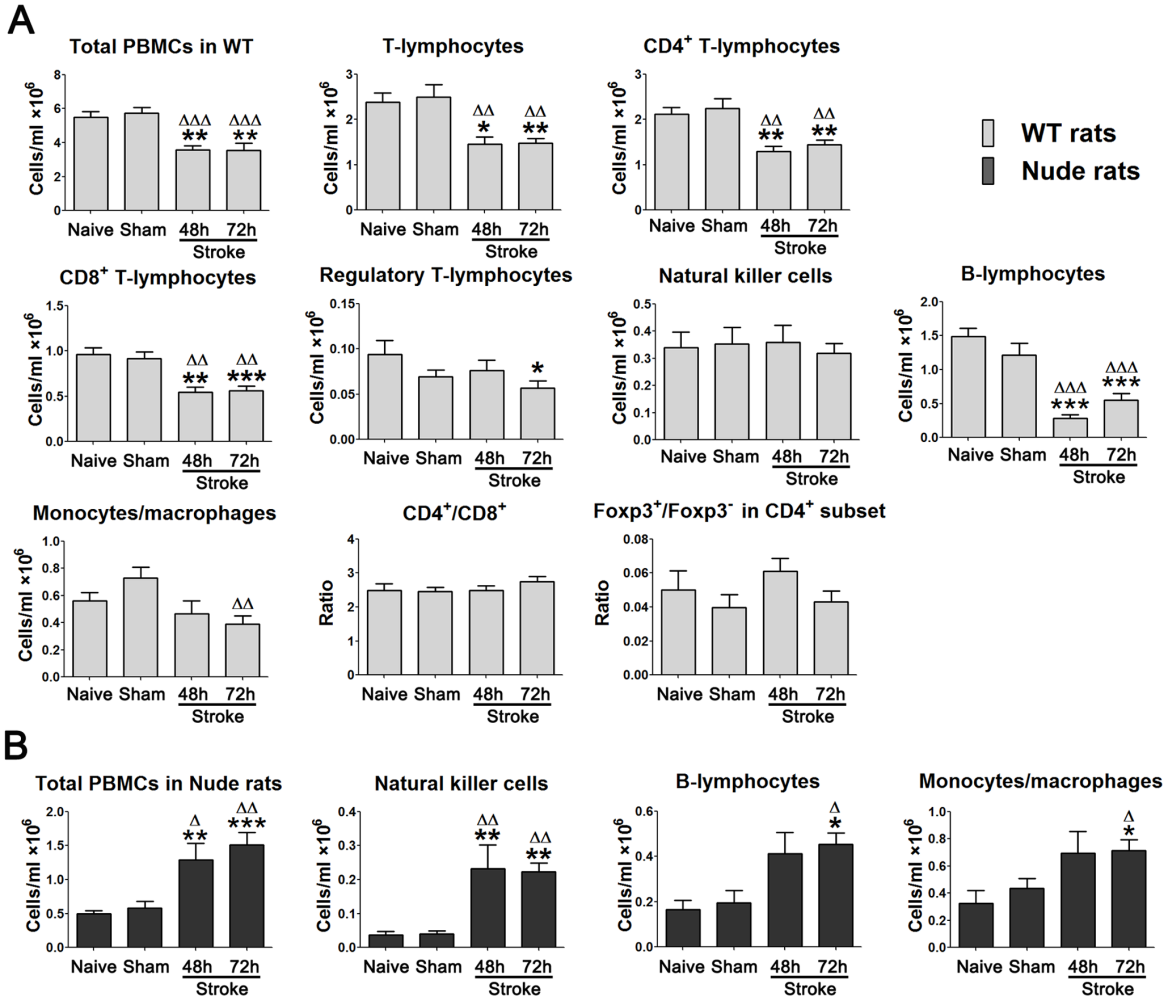
Total number of PBMCs and immune cell subsets in the peripheral blood of WT and nude rats. A. Total number of PBMCs and absolute numbers of T cells, CD4^+^ T cells, CD8^+^ T cells, Tregs, B cells, NK cells, monocytes, CD4^+^/CD8^+^ as well as the Foxp3^+^/Foxp3^−^ ratio in CD4^+^ T cells in SD WT rats (n = 5–11/group). B. Total number of PBMCs, absolute numbers of B cells, NK cells and monocytes in nude rats (n = 5–8/group), Blood was harvested from the tail 48 h or 72 h after stroke and total PBMCs were isolated for immunostaining. Relative percentages of each cell type were obtained from the FACS analyses using PBMCs. Absolute cell numbers were calculated by multiplying the percentage of each cell type with the total cell number in an analyzed sample, and adjusted to a number in 1 ml of blood. For naïve groups, no surgeries were performed. For sham groups, rats received sham surgery without stroke. *, **, *** vs. naïve, ^Δ^, ^ΔΔ^, ^ΔΔΔ^ vs. sham, p<0.05, 0.01, 0.001, respectively.

Flow cytometry was used to measure immune cell subsets. Gating strategies for each immune cell type are shown (Fig. 3). Cell types of T cells, CD4^+^ T cells, CD8^+^ T cells, Treg cells, NK cells, and monocytes/macrophages were analyzed. There are many forms of regulatory cells, but in this study, the best understood CD4^+^CD25^+^FoxP3^+^ cell was examined. We first counted total PBMCs in naïve, sham surgery and stroke rats at 48 and 72 h post stroke (Fig. 4). As nude rats are athymic and have no T cells, there are considerably fewer circulating immune cells in nude rats than in WT rats. The results show that stroke, but not sham surgery, resulted in a significant decrease in total PBMCs in WT rats at both 48 and 72 h, which is an SIID hallmark after stroke (Fig. 4A). However, to our surprise, total PBMCs were significantly increased after stroke in T-cell-deficient nude rats compared to non-stroke nude rats (Fig. 4B), suggesting that T cells are critical for SIID induction.

We then calculated the cell numbers for immune cell subsets in PBMCs using FACS analyses in both WT and nude rats (Fig. 4). Stroke resulted in a significant reduction in absolute numbers of T cells, CD4^+^ T cells, CD8^+^ T cells, Tregs, B cells, and monocytes, either at 48 h or at 72 h, or both. However, NK cell numbers were not reduced in WT rats receiving stroke compared with naïve and sham surgery rats. The ratios of CD4^+^/CD8^+^ and Foxp3^+^/Foxp3^−^ in CD4^+^ cells were not altered in stroke animals compared with naïve and sham surgery rats (Fig. 4A). In contrast to WT rats, numbers of B cells, NK cells, and monocytes in T-cell-deficient nude rats were robustly increased after stroke compared with both naïve and sham surgery (Fig. 4B). Note that nude rats, in principle, have no T cells, including CD4^+^ T cells, CD8^+^ T cells and Tregs as these T cells must differentiate and mature in the thymus, which nude rats lack. These results suggest that reductions in non-T immune cells in WT rats are T cell dependent.

### Reductions in splenocytes occurred in WT but not in nude rats after stroke

**Figure 5:**
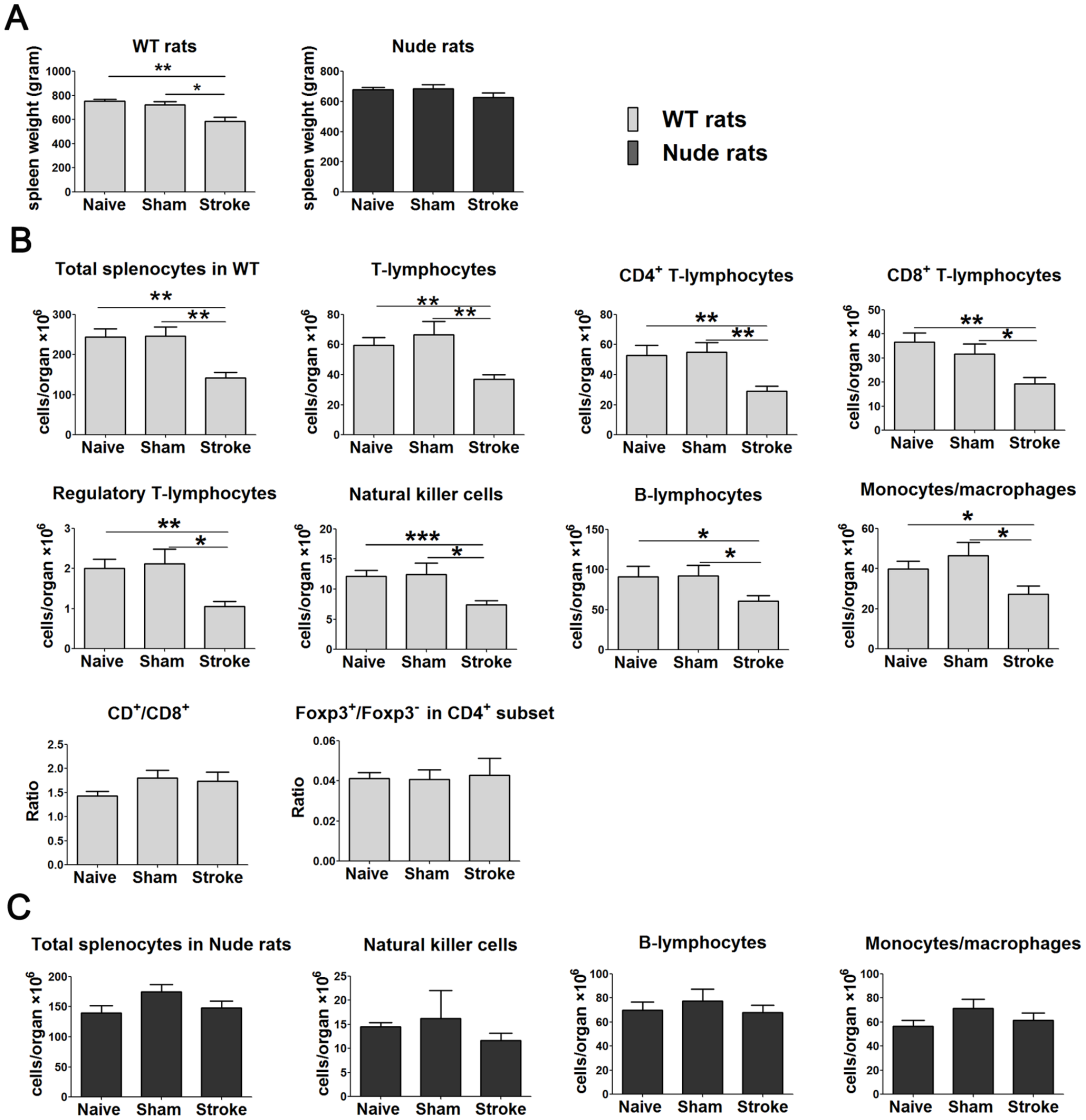
Spleen size, total number of splenocytes and individual subsets in WT and nude rats. A. Spleen weight (n = 5–6/group). B. Total number of splenocytes, absolute numbers of T cells, CD4^+^ T cells, CD8^+^ T cells, Tregs, B cells, NK cells, monocytes, CD4^+^/CD8^+^ and Foxp3^+^/Foxp3^−^ ratio in CD4^+^T cells in WT SD rats (n = 5–11/group). C. Total number of splenocytes and absolute numbers of B cells, NK cells as well as monocytes in nude rats (n = 5–8/group). Spleen was collected 3 d after stroke and total splenocytes isolated for immunostaining. Relative percentages of each cell type were obtained from the FACS analyses using splenocytes. Absolute cell numbers were calculated by multiplying the percentage of each cell type with the total cell number in an analyzed sample, and adjusted to a number in 1 spleen. *, **, *** vs. stroke, p<0.05, 0.01, 0.001, respectively.

We then measured the spleen weight and the total number of splenocytes and absolute number of cell subpopulations in the spleen of naïve, sham surgery and stroke rats 72 h post-stroke (Fig. 5). Spleen weight was significantly lost after stroke in WT rats but not in the nude rats (Fig. 5A). Stroke in WT rats induced significant decreases in total numbers of splenocytes and absolute numbers of all measured immune-cell subsets, including T cells, CD4^+^ T cells, CD8^+^ T cells, Tregs, NK cells, B cells and monocytes (Fig. 5B). In contrast, total numbers of splenocytes and absolute numbers of B cells, NK cells, and monocytes were not significantly altered in nude rats received stroke compared with both naïve and sham surgery (Fig. 5C). Like in PBMC, in the ratios of CD4^+^/CD8^+^ T cells and Foxp3^+^/Foxp3^−^ in CD4^+^ cells were not changed in the spleen in WT rats after stroke (Fig. 5B). Again, these results suggest that the existence of T cells is critical for spleen atrophy characterized by loss of splenocytes.

### Adoptive transfer of WT splenocytes to nude rats generated lymphopenia induced by stroke

**Figure 6:**
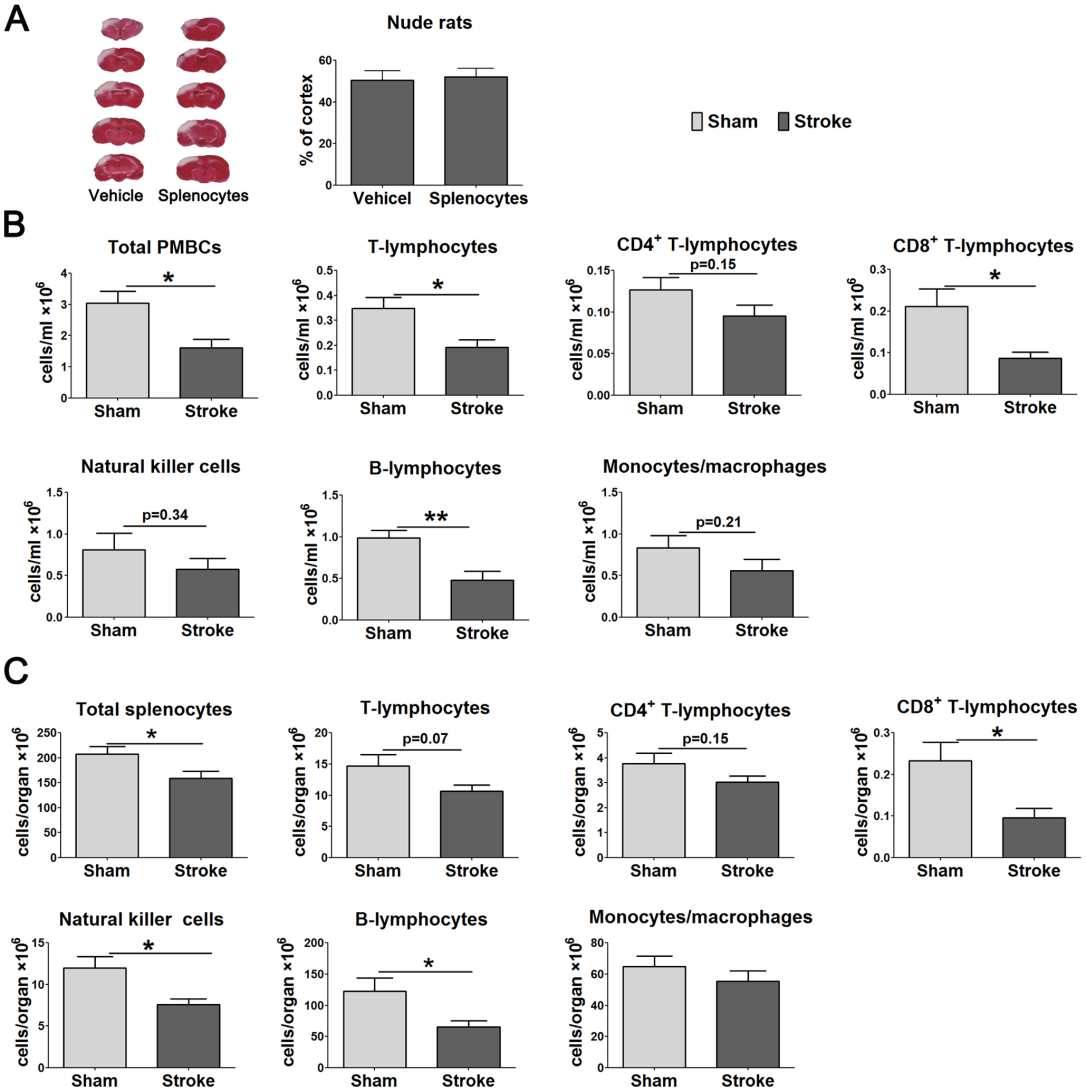
Infarct size and numbers of PBMCs, splenocytes, and individual cell subsets in nude rats receiving adoptive lymphocyte transplant. Nude rats were reconstituted by intravenous injection of 1×10^8^ splenocytes from WT rats and stroke was conducted 24 h later. Infarct sizes, PBMCs and splenocytes were measured 3 days after stroke. A. Comparison of infarct sizes between vehicle and lymphocyte reconstitution group in nude rats (n = 6/group). B. Total number of PBMCs and absolute numbers of T cells, CD4^+^ T cells, CD8^+^ T cells, B cells, NK cell, as well as monocytes in the blood in reconstituted nude rats (n = 6–7/group). C. Total number of splenocytes, absolute numbers of T cells, CD4^+^ T cells, CD8^+^ T cells, B cells, NK cell and monocytes in the spleen in reconstituted nude rats (n = 6–7/group). *, ** vs. sham, p<0.05, 0.01, respectively.

If decreases in non-T immune cells are attributable to T cells, the reconstitution of T cells in nude rats should produce lymphopenia. We injected 10^8^ splenocytes from WT rats via the external jugular vein into nude rats and induced stroke 24 h later. Infarct sizes, total numbers of PBMCs and splenocytes, as well as the absolute numbers of immune cell subtypes were analyzed 3 d after stroke. Results showed that infarct sizes do not differ between nude rats receiving WT splenocytes and vehicle (Fig. 6A). However, total numbers of PBMCs and splenocytes were reduced in nude rats receiving splenocyte transfer and stroke compared with nude rats receiving splenocyte transfer but without stroke (Fig. 6B–C). In addition, absolute numbers of T cells, CD8^+^ T cells and B cells were significantly reduced after stroke in the peripheral blood of nude rats receiving splenocyte transfer and stroke; the number of CD4^+^ T cells, NK cells and monocytes also showed a reduction trend, though significance was not reached (Fig. 6B). Furthermore, stroke also resulted in significantly reduced CD8^+^ T cells, NK cells and B cells in the spleen, and with insignificantly-reduced T cells, CD4^+^ T cells and monocytes in the spleens of nude rats that received splenocyte adoptive transfer (Fig. 6C).

### Stroke resulted in inhibition of in vitro T cell proliferation of WT rats as well as nude rats after WT splenocyte adoptive transfer

**Figure 7:**
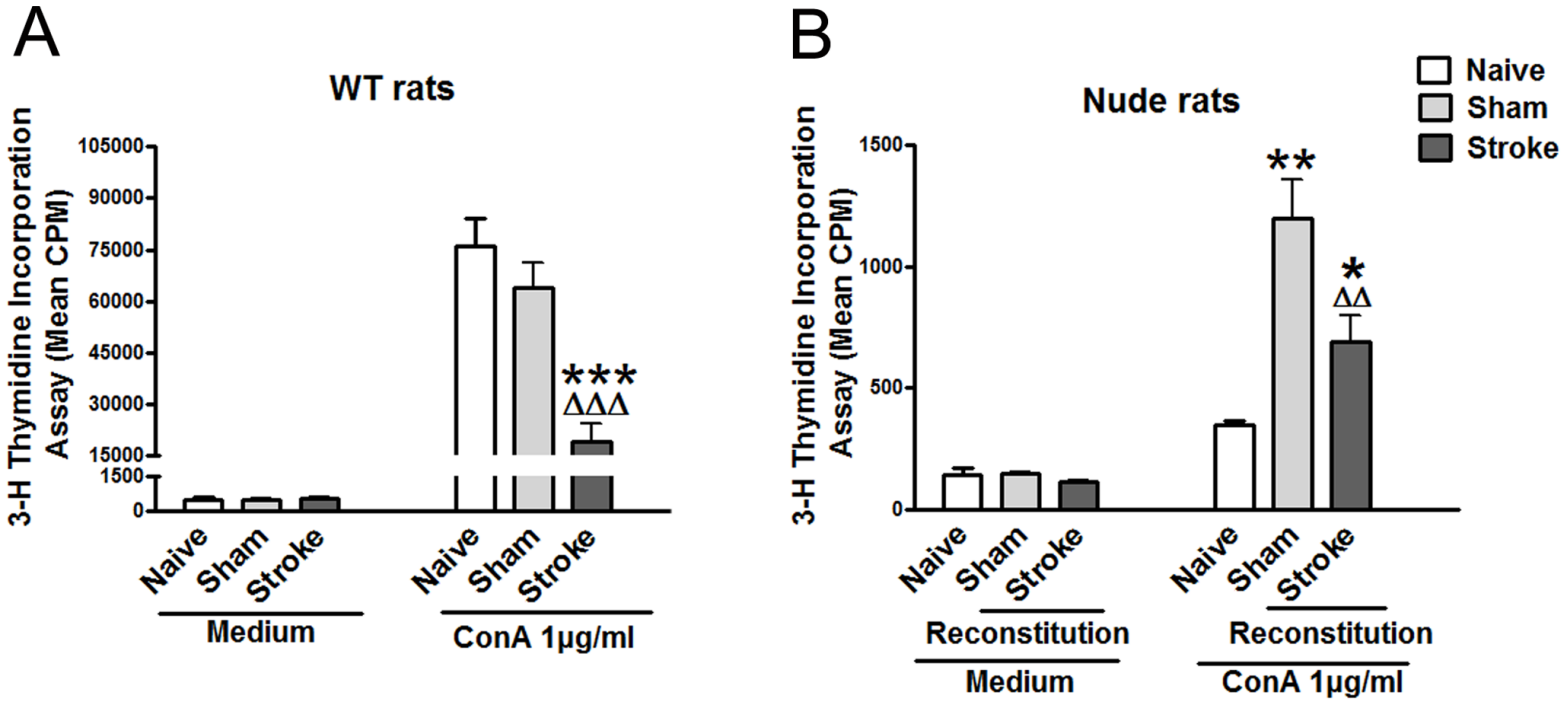
T cell proliferative assay after stroke. Splenocytes were prepared 72 h after stroke from naïve, sham surgery and stroke animals. Cultured splenocytes were stimulated with 0 μg/ml, 1 μg/ml Con A. The cells were incubated for 66 h at 37°C and 5% CO_2_. [^3^H]-Thymidine was added to the culture and incubated for 18 h. Cells were harvested onto a glass fiber filter. The amount of radioactivity that integrated into divided lymphocytes was measured by a beta-plate scintillation reader. Results are expressed as counts per minute (cpm). A. Splenocytes proliferation in WT SD rats (n = 3–6/group). B. Splenocytes proliferation in T cells reconstituted nude rats (n = 3–5/group). Nude rats were reconstituted by intravenous injection of 1×10^8^ splenocytes in 100 μl RPMI 1640, and dMCAO was performed 24 h after reconstitution. *, **, *** vs. naïve, ^Δ^, ^ΔΔ^, ^ΔΔΔ^ vs. sham, p<0.05, 0.01, 0.001, respectively.

To confirm that stroke-induced lymphopenia corresponds to inhibited immune function, we further evaluated the proliferative ability of splenocytes stimulated by Con A from WT rats and nude rats that received splenocytes. Results showed that proliferation of T cells was significantly inhibited by stroke in WT rats as well as in nude rats after WT splenocyte adoptive transfer (Fig. 7). We noticed that the overall proliferative activity of T cells is much lower in nude rats receiving splenocytes than in WT rats. This is because only 10^8^ splenocytes was transferred to nude rats, and this number is much lower than the total splenocytes of a WT rat. In addition, these cells were injected into a vein, thus not all of them could accumulate in the spleen. As a result, the measured-T cell proliferative activity is low in nude rats receiving adoptive splenocyte transfer.

### HMGB1 release in the plasma is associated with lymphopenia

**Figure 8:**
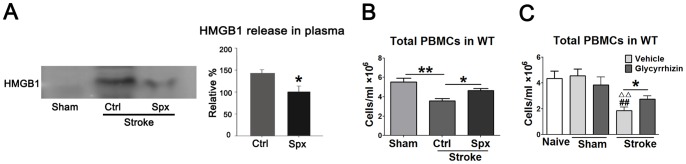
HMGB1 levels in the plasma and corresponding total number of PBMCs after splenectomy or treatment with Glycyrrhizin followed by stroke. A. Western blot detected HMGB1 release in the plasma 48 h after stroke in rats with and without splenectomy. The left panel shows representative results of Western blot for HMGB1. Note that no control protein, such as β-actin, was used to show even loading, because the examined samples are plasma, for which the same amount volume (15 μl) from different animals was loaded. The bar graphs in the right panel show the average optical density of protein bands. The relative density of protein bands in rats receiving splenectomy plus stroke was set as 100%. * vs. control (stroke without splenectomy), P<0.05, n = 4/group. B. Total number of PBMCs in stroke rats with or without splenectomy. Forty-eight hours after stroke followed splenectomy, blood was harvested and total PBMCs were isolated and counted. Splenectomy significantly attenuated reduction in total number of PBMCs induced by stroke. *, ** vs. control, p<0.05, 0.01, respectively. N = 9–11/group. Ctrl: Control; Spx: splenectomy. C. Total number of PBMCs in stroke rats treated with or without glycyrrhizin. Glycyrrhizin (200 mg/kg) was injected intraperitoneally immediately before stroke and 2 h after reperfusion. The blood was harvested and total PBMCs were isolated and counted 3 d post stroke. Glycyrrhizin significantly reversed the reduction in total PBMCs induced by stroke in vehicle. ^##^ vs. naïve, ^ΔΔ^, vs. Sham, p<0.01, * vs. vehicle, p<0.05. N = 9–11/group.

HMGB1 is a cytokine-like protein, which regulates both inflammatory response and immunodepression. We used western blot to measure HMGB1 levels in the peripheral blood 48 h after stroke onset, and found that HMGB1 was released in the plasma in WT rats but not in nude rats (data not shown), suggesting that HMGB1 release is associated with T cells. In addition, splenectomy is known to reduce infarction after stroke [33], and it is known to increase lymphocyte numbers in the peripheral circulation in both patients and animals [36], [37]. Our pilot study showed that splenectomy attenuated lymphopenia in the blood (Fig. 8B), thus we further examined if this is related with less HMGB1 being released in the plasma. Indeed, our data showed that splenectomy reduced HMGB1 release in the plasma (Fig. 8A), which suggests a correlation between the reduction in HMGB1 release and attenuation in lymphopenia after splenectomy in stroke. Furthermore, Glycyrrhizin, a HMGB1 inhibitor, significantly attenuated reductions in PBMC numbers 3 d after stroke (Fig. 8C).

## Discussion

Ours is the first report to demonstrate that stroke increases total PBMCs as well as B cells, NK cells and monocytes in T-cell-deficient nude rats. In addition, total numbers of splenocytes in nude rats were not reduced after stroke. These results strongly contrast with the considerable post-stroke lymphopenia in the peripheral blood and spleen of WT rats. As WT splenocyte adoptive transfer to nude rats produced stroke-induced lymphopenia, our data suggest that in peripheral organs, T cells regulate decreases in non-T immune cells, including B cells, NK cells and monocytes. Furthermore, our data suggests that HMGB1 is associated with T cell-mediated lymphopenia after stroke.

SIID, characterized by lymphopenia, is a well-known phenomenon in clinical stroke patients and experimental stroke animals [10], [38], [39], [40] that starts a few hours after stroke and may last a few months. Stroke has also been shown to greatly inhibit in vitro proliferation of T cells in response to antigen stimulation [4], [5], [11]. These studies used either clinical blood samples from human stroke patients or blood samples and peripheral lymphoid organs from mouse stroke models [4], [5], [11]. Our study confirms that stroke can also produce SIID in a rat stroke model, as evidenced by lymphopenia in the peripheral blood and spleen as well as inhibited T cell proliferation in vitro.

Although we demonstrate that T cells regulate non-T immune cell populations after stroke, future studies will be necessary to identify which T cell subsets are most central to immunosuppression. T cells are classified as CD4^+^ (helper T cells, Th) and CD8^+^ (cytotoxic T cells, CTL) T cells, according to their surface expression of CD4 and CD8 molecules [41]. Functionally, Th cells are further classified as Th1, Th2, Th17, and Tfh cells, based on their cytokine secretion profiles [42], [43], [44]. Another CD4^+^ T cell type, regulatory T cell (Tregs) expresses transcription factor Foxp3, which is important for the maintenance of immunologic tolerance [45], [46], [47]. Most recently, we have demonstrated that T cell subsets play different roles in brain injury induced by stroke [31]. We showed that the lack of CD4^+^, CD8^+^ T cells, or total T cells, resulted in smaller infarction measured 2 d post-stroke. In addition, while Th1 deficiency led to smaller infarction, Th2 deficiency resulted in larger infarction, but Treg deficiency had no impact on acute infarction measured 2 d after stroke [31]. In our current study, CD4^+^ T cells, CD8^+^ T cells and Tregs all decreased. To determine how distinctive T cell subsets correlate with peripheral immunosuppression requires further study by using genetic animals with T cell subset deficiencies and antibodies to delete specific T cell types.

The underlying mechanisms of SIID are not completely understood. Prass and colleagues were the first to report that SIID is likely induced by activation of the SNS [4]. They found that stroke produces long-lasting lymphopenia and impaired cytokine expression in the spleen, thymus and peripheral blood, which causes spontaneous septicemia, pneumonia and mortality in a mouse stroke model. Administration of the β-adrenoreceptor blocker propranolol greatly attenuated mortality after stroke, so the SNS appears to play a critical role in SIID [4]. Most recently, this theory was further extended by Wong et al., who reported that stroke-induced SNS activation alters the behavior and function of iNKT cells, a major subset of NKT cells that is responsible for SIID [17]. In addition to these theories, Offner and colleagues measured an acute pro-inflammatory response between 6 h and 22 h post-stroke, which had shifted to an anti-inflammatory response, i.e., immunosuppression, by 96 h post-stroke [5]. They suggest that SIID is likely induced by increases in Tregs, which are known to be anti-inflammatory [5]. Our innovative findings suggest that T cells regulate the reduction of non-T immune cells, including B cells, NK cells and monocytes, and that HMGB1 may play a critical role in this process. Stroke may first result in T cell dysfunction within peripheral organs, as we have shown the ability of T cell proliferation was greatly inhibited after stroke. In addition, stroke leads to the massive apoptosis of T cells, which has been demonstrated by several previous studies that large apoptotic cells are presented in the spleen and thymus after stroke [4], [5], [39]. Stroke also results in a shift from pro-inflammatory Th1 to anti-inflammatory Th2 response in animal stroke models [4] and a significantly lower ratio of IFNγ/IL-4-producing T cells in human patients [48]. Dysfunctional T cells or T cells with altered functions might then secrete more immunosuppressive factors such as IL-4 and IL-10, or regulate the release of inflammatory/immunodepressive factors from other cell types that result in reductions in B cells, NK cells and monocytes cells. In this study, we found that HMGB1 was released in the peripheral blood of WT rats but not of nude rats, suggesting that T cells mediate HMGB1 release. It is well-known that HMGB1 is a cytokine-like protein, which is released into the CSF and peripheral blood after stroke [22], [23]. It has dual roles: when released in the necrotic tissue, it promotes inflammatory response [23], [24], [25]; but when released into the peripheral blood, it may cause immunodepression [26]. Since HMGB1 was only detected in the plasma of WT rats with T cells but not in that of nude rats without T cells, it is plausible to speculate that T cells mediate HMGB1 release. Some other studies have shown that HMGB1 promotes immunodepression by either directly enhancing Treg cell functions [26], [49] or interacting with Toll-like receptor (TLR) 4 on dendritic cells (DC) or on Treg cells to promote immunosuppression [50], [51]. Based on these previous findings and our own results, it is likely that HMGB1 is a critical factor mediated by T cells that lead to reductions in other non-T cell lymphocytes. In fact, our further evidence showed that the amount of HMGB1 release in the plasma conversely correlates with PBMC numbers in animals with and without receiving splenectomy. Splenectomy is known to reduce infarction after stroke [33], but it is also known to increase circulating lymphocyte numbers in the absence of stroke [36], [37]. We hypothesized that splenectomy attenuates lymphopenia after stroke. Indeed, we found that PBMC numbers were increased in rats with splenectomy after stroke, and this is associated with less HMGB1 release in the blood. Furthermore, we showed that glycyrrhizin, as a HMGB1 inhibitor, which directly combines with HMGB1 and induces conformational changes, significantly attenuated lymphopenia. Taken together, our results indicate a potential role of HMGB1 in regulating the effects of T cells in stroke-induced lymphopenia. However, more studies are required to address how HMGB1 is released through T cell-mediated effects, and how released HMGB1 affects the fate of other non-T cell lymphocytes.

Although SIID is described consistently in most studies, including its effects on lymphopenia and inhibited T-cell-mediated immunity, controversial results remain. For instance, the effect of stroke on B-cell-mediated humoral immunity is debatable. An early study by Czlonkowska et al. showed that after stroke, IgM and IgG immunoglobulins were unchanged while IgA was increased [7]. Urra et al. also found that B cells were decreased along with T, Th, and CTL cells, and that poor outcome was associated with reduced B cells [12]. In contrast, Vogelgesang et al. found that B cells were unaffected whereas CD4^+^ and CD8^+^ lymphocytes decreased [10]. Furthermore, Yan et al. showed that activated B cells increased [52]. Nevertheless, all animal studies, including our current study, suggest that B cells are dramatically reduced after stroke. Similar controversies persist as to whether or not NK cells and Tregs, as well as monocytes, increase after stroke [4], [5], [10], [11], [19], [20], [21]. Our study shows that every subset, except NK cells, was reduced after stroke in WT rats. Considering that Wong et al. recently showed that iNKT cells, a major subset of NKT cells, play a critical role in SIID, these controversies may not be minor issues. More research is therefore needed to address the functions of non T-cell subsets in SIID.

In conclusion, we demonstrated that total lymphocyte numbers as well as lymphocyte subsets and monocytes in peripheral blood increased after stroke in T-cell-deficient nude rats but decreased in WT SD rats and in nude rats after T cell reconstitution. Moreover, stroke induced similar inhibition in the proliferation of splenocytes in nude rats after adoptive splenocyte transfer and in SD WT rats in vitro. Our study suggests that T cells regulate reduction in non-T immune cells after stroke.
